# Reactive Oxygen Species Induces Lipid Droplet Accumulation in HepG2 Cells by Increasing Perilipin 2 Expression

**DOI:** 10.3390/ijms19113445

**Published:** 2018-11-02

**Authors:** Yi Jin, Yanjie Tan, Lupeng Chen, Yan Liu, Zhuqing Ren

**Affiliations:** 1 Key Laboratory of Agriculture Animal Genetics, Breeding and Reproduction of Ministry of Education, College of Animal Science, Huazhong Agricultural University, Wuhan 430070, China; hyj_1900@webmail.hzau.edu.cn (Y.J.); tanyanjie@webmail.hzau.edu.cn (Y.T.); chenlupeng@webmail.hzau.edu.cn (L.C.); liu-yan@webmail.hzau.edu.cn (Y.L.); 2 The Cooperative Innovation Center for Sustainable Pig Production, Huazhong Agricultural University, Wuhan 430070, China

**Keywords:** NAFLD, ROS, PLIN2, lipid droplet

## Abstract

Non-alcoholic fatty liver disease (NAFLD) has become the world’s most common liver disease. The disease can develop liver fibrosis or even carcinomas from the initial hepatic steatosis, and this process is influenced by many factors. Reactive oxygen species (ROS), as potent oxidants in cells, have been reported previously to play an important role in the development of NAFLD progression via promoting neutral lipid accumulation. Here, we found that ROS can promote lipid droplet formation in hepatocytes by promoting perilipin2 (PLIN2) expression. First, we used different concentrations of hydrogen peroxide to treat HepG2 cells and found that the number of lipid droplets in the cells increased, however also that this effect was dose-independent. Then, the mRNA level of several lipid droplet-associated genes was detected with hydrogen peroxide treatment and the expression of *PLIN2*, *PLIN5*, and *FSP27* genes was significantly up-regulated (*p* < 0.05). We overexpressed *PLIN2* in HepG2 cells and found that the lipid droplets in the cells were markedly increased. Interference with *PLIN2* inhibits ROS-induced lipid droplet formation, revealing that PLIN2 is a critical factor in this process. We subsequently analyzed the regulatory pathway and protein interaction network that is involved in PLIN2 and found that PLIN2 can regulate intracellular lipid metabolism through the PPARα/RXRA and CREB/CREBBP signaling pathways. The majority of the data indicated the correlation between hydrogen peroxide-induced PLIN2 and lipid droplet upregulation. In conclusion, ROS up-regulates the expression of PLIN2 in hepatocytes, whereas PLIN2 promotes the formation of lipid droplets resulting in lipid accumulation in liver tissues.

## 1. Introduction

Non-alcoholic fatty liver disease (NAFLD) is a metabolic syndrome of the liver that affects a large number of patients worldwide. Unhealthy lifestyles and diets greatly increase the prevalence of obesity and type 2 diabetes, and these two types of disease often increase the incidence of NAFLD. A typical feature of NAFLD is the accumulation of large amounts of fat (mainly triglycerides) in the liver tissue. The disease includes non-alcoholic hepatitis (NASH), chronic interstitial hepatitis, hepatic failure, and even hepatocellular carcinoma [1,2,3]. NAFLD is primarily characterized by disorders of lipid metabolism. The occurrence and development of NAFLD are affected by many factors. One such important factor is oxidative stress; however, existing studies have not yet established the causal relationship between oxidative stress and NAFLD [1,4,5]. Studies have demonstrated that reactive oxygen species (ROS) levels in the liver tissues of patients with NAFLD and NASH are increased, and the expression levels of superoxide dismutase (SOD) and other antioxidant enzymes are decreased [6]. Elevated levels of ROS affect the homeostasis of intracellular lipid metabolism, causing disturbances in lipid synthesis and decomposition processes. For example, an increase in ROS levels in glial cells leads to decreases in mitochondrial activity and fatty acid oxidation decomposition levels, while higher ROS levels further activate c-Jun-N-terminal kinase (JNK) and sterol regulatory element binding protein 1c (SREBP1c), resulting in neutral lipid aggregation [7]. In addition, loss of endogenous antioxidant enzymes or exogenous addition of ROS will lead to mitogen-activated protein kinase (AMPK) and sterol-regulatory element binding proteins (SREBP) pathways and promote neutral lipid production [8,9,10]. In terms of lipid breakdown, it has been reported that ROS have an important regulatory role in the recruitment of lipase on lipid droplet surfaces and in the recognition and degradation of lipid droplets by autophagic vacuoles [11,12]. A recent study has showed that high ROS would induce endoplasmic reticulum (ER) stress which promotes the lipid accumulation to aggregate the development of NAFLD and NASH [13]. Moreover, ROS also plays an important role in alcoholic liver disease and hepatocellular carcinoma via regulating the AMPK signaling pathway [14]. One important reason for the increase of ROS is more free fatty acid intake or release. A study about lipodystrophies showed that an impaired ability of lipid storage induced oxidative stress and lipotoxity [15]. Therefore, ROS levels have an influential effect on the homeostasis of lipid metabolism in hepatocytes, however its specific regulatory mechanism is not yet clear.

Intracellular lipids are mainly stored in lipid droplets. As unique organelles, lipid droplets play an important role in the regulation of lipid metabolism and are the center of cellular lipid metabolism. The perilipin, ADRP, TIP47 (PAT) protein family plays an important role in lipid droplet formation. Five members of the PAT family have now been found, namely perilipin1, perilipin2, perilipin3, perilipin4, and perilipin5 (PLIN1-PLIN5). PAT proteins contain an amphipathic helical structure with large hydrophobic residues that can bind tightly to the lipid droplet surface [16]. Among them, PLIN1, PLIN3, and PLIN5 are related to the oxidative breakdown of lipids. PLIN1 can interact with ATGL and CGI-58 to regulate triglyceride hydrolysis [17]. PLIN1 can also bind to Mnf2, stabilizing lipid droplets and mitochondrial contact [18]. PLIN3 regulates intracellular lipid degradation and fatty acid oxidation [19,20]. PLIN5 plays a key role in the interaction of lipid droplets with mitochondria and directly regulates the oxidative metabolism of neutral lipids in lipid droplets [21,22,23,24]. PLIN2 is of great importance to the formation of intracellular lipid droplets. Interfering with PLIN2 results in a significant decrease in the number and diameter of intracellular lipid droplets [25]. PLIN2 blocks the contact of lipase with neutral lipids, thereby inhibiting the hydrolysis of triglycerides and maintaining the stability of lipid droplets [26,27]. In addition, PLIN2 plays an important role in steatosis of hepatocytes. *PLIN2* knockout mice do not respond to diet-induced obesity, fatty inflammation, and hepatic steatosis [28,29,30,31]. The immunohistochemical analysis showed that PLIN2 localized to the surface of lipid droplets in fatty liver tissues, which indicated that PLIN2 was involved in the progression of fatty liver disease [32]. However, little is known about whether elevated reactive oxygen species in NAFLD influence the expression of lipid homeostasis in cells by regulating the expression of PAT family members.

In the present study, we found that increased levels of reactive oxygen species promoted the expression of the *PLIN2* gene and increased the lipid droplet content in HepG2 cells through the PPARα/RXRA and CREB/CREBBP pathways. Our study attempts to provide evidence for the role of ROS and oxidative stress in the development of NAFLD, providing clues for the molecular mechanisms of NAFLD progress and progression.

## 2. Results

### 2.1. Exogenous Addition of Hydrogen Peroxide Promotes the Formation of Lipid Droplets in Cells

**Figure 1:**
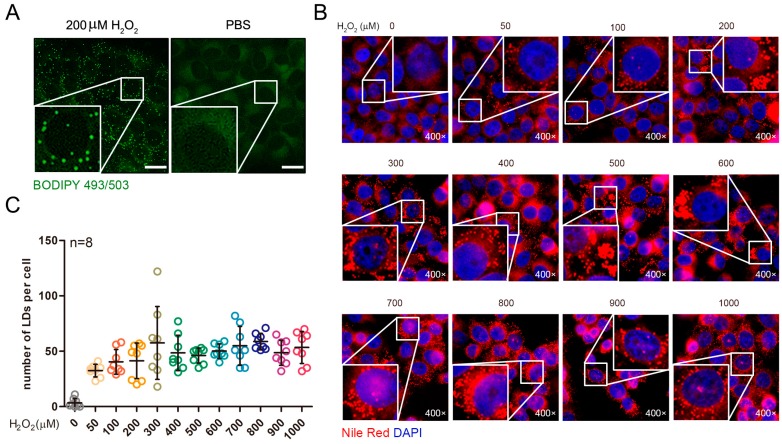
Increased lipid droplets in HepG2 cells after hydrogen peroxide treatment were not dose dependent. (**A**) The cells were labeled with lipid droplets after treatment with 200 μM hydrogen peroxide. Scale bar = 10 μm. (**B**) Labelling of the intracellular lipid droplets after treatment of the cells with different concentrations of hydrogen peroxide. (**C**) Statistics on the number of lipid droplets in the cell.

According to previous studies, reactive oxygen species in cells contain various forms of active oxygen ions and hydrogen peroxide. Hydrogen peroxide can easily penetrate the cell membrane and the ROS molecules in the cytoplasm are mainly hydrogen peroxide [33]. Therefore, in order to detect the effect of reactive oxygen species on the formation of intracellular lipid droplets, we exogenously added hydrogen peroxide to the cell culture solution, mimicking the state of intracellular reactive oxygen increase. Drawing upon previous experience in our lab, the hydrogen peroxide treatment concentration was first set at 200 μM, and the cells were treated for 8 h. The control group was treated with an equal volume of PBS (phosphate buffer saline) buffer. After treatment, intracellular lipid droplets were labeled with BODIPY 493/503 and were observed under a fluorescence microscope. The results showed that there were significantly more lipid droplets in the treated group than in the control group (Figure 1A).

ROS concentration in cells changes dynamically; different concentrations of ROS can have different effects on the formation of intracellular lipid droplets. Therefore, we established a concentration gradient and used different concentrations of hydrogen peroxide to treat cells with respect to intracellular lipids. The drops were labeled, and their number was counted (Figure 1B), and the effect of hydrogen peroxide concentration on the number of lipid droplets in the cells was analyzed. Hydrogen peroxide treatment concentrations were 50, 100, 150, 200, 250, 300, 350, 400, 450, 500, and 1000 μM; no hydrogen peroxide was added to the control group. The results showed that all of the concentrations of hydrogen peroxide promoted the formation of intracellular lipid droplets, however different concentrations had no significant effect on the number of intracellular lipid droplets (Figure 1C).

### 2.2. PLIN2 Expression Level Increased after Treatment by Hydrogen Peroxide

**Figure 2:**
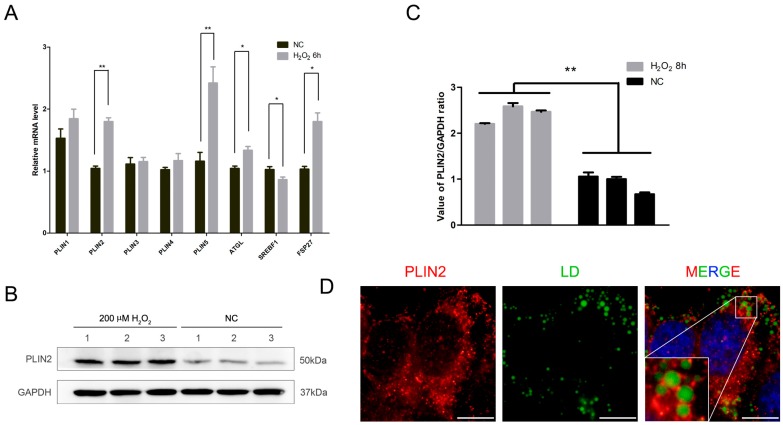
Hydrogen peroxide promotes the expression of PLIN2 (perilipin2). (**A**) Detection of lipid droplet-related gene mRNA expression levels after hydrogen peroxide treatment. *, *p* < 0.05; **, *p* < 0.01. (**B**) Western blot analysis of PLIN2 protein expression levels. (**C**) Density analysis of PLIN2 protein expression levels. **, *p* < 0.01. (**D**) Cellular immunofluorescence detects the localization of PLIN2 in cells. Scale bar = 10 μm.

We observed that hydrogen peroxide could promote the formation of intracellular lipid droplets. Lipid droplet generation is a complex process and there are many factors involved in it; existing research is not able to fully explain the process of lipid droplet formation. Based on previous studies, we selected several genes related to lipid droplet production to investigate the changes of lipid droplet-associated gene expression after hydrogen peroxide treatment. The cells were treated with 200 μM hydrogen peroxide for 6 h. The treated and control cells were collected, and total RNA was extracted to prepare a cDNA library. We detected the relative mRNA expression levels of *PLIN1*, *PLIN2*, *PLIN3*, *PLIN4*, *PLIN5*, *ATGL*, *SREBP1*, and *FSP27* (Figure 2A). The results showed that the expression levels of *PLIN2*, *PLIN5*, *ATGL*, and *FSP27* were increased significantly (*p* < 0.05), whereas *SREBP1* expression decreased significantly (*p* < 0.05). We further examined the protein expression level of *PLIN2* and found that it was significantly increased (*p* < 0.05) (Figure 2B,C). Since hydrogen peroxide treatment results in elevated *PLIN2* expression, it is necessary to understand the location of the gene expression product; thus, we located its enriched position in cells by means of cellular immunofluorescence. The results showed that PLIN2 is mainly recruited on the surface of lipid droplets (Figure 2D).

### 2.3. PLIN2 Overexpression Promotes Lipid Droplets Formation in Cells

**Figure 3:**
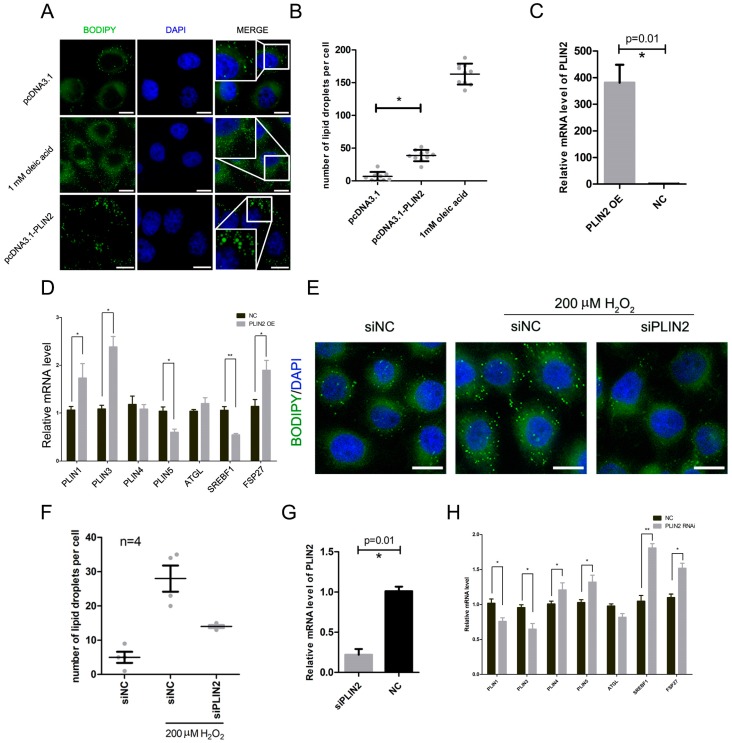
PLIN2 plays an important role in ROS (reactive oxygen species)-induced lipid droplet formation. (**A**) The amount of intracellular lipid droplets detected after overexpression of PLIN2. Scale bar = 10 μm. (**B**) Statistical analysis of the number of lipid droplets. *, *p* < 0.05. (**C**) qRT-PCR detection of the efficiency of *PLIN2* overexpression. *, *p* < 0.05. (**D**) Detection of lipid droplet-related gene mRNA expression levels with overexpression of *PLIN2*. *, *p* < 0.05; **, *p* < 0.01. (**E**) Measurement of the amount of lipid droplets in cells treated with hydrogen peroxide after interference with PLIN2. Scale bar = 10 μm. (**F**) Statistical analysis of lipid droplet numbers. (**G**) qRT-PCR detection of the interference efficiency of PLIN2. *, *p* < 0.05. (**H**) Detection of lipid droplet-related gene mRNA expression levels after interference with *PLIN2*. *, *p* < 0.05; **, *p* < 0.01.

In order to detect the increase of intracellular lipid droplets caused by the increased expression of *PLIN2*, we constructed a pcDNA3.1-PLIN2 eukaryotic expression vector, overexpressed *PLIN2* in HepG2 cells, and detected the number of intracellular lipid droplets. The pcDNA3.1-PLIN2 vector was transfected into cells (pcDNA3.1 empty vector in the negative control group and 1 mM oleic acid in the positive control group) using Lipofectamine 2000, and the cells were further cultured for 48 h to ensure that the vector was fully expressed in the cells. The transfection efficiency of the cells was determined using the pCMV-mCherry-C1 vector. The intracellular lipid droplets were labeled with BODIPY 493/503 and were observed under a fluorescence microscope. The results showed that the number of lipid droplets in the overexpression group was significantly increased compared to the control group (*p* < 0.05) (Figure 3A,B).

We measured the relative expression of *PLIN2* to determine the effect of overexpression and the result showed that the expression level of *PLIN2* was significantly increased (*p* < 0.05) (Figure 3C). We further examined the expression levels of *PLIN1*, *PLIN3*, *PLIN4*, *PLIN5*, *ATGL*, *SREBP1*, and *FSP27*. The results showed that the expression levels of *PLIN1*, *PLIN3*, and *FSP27* increased significantly (*p* < 0.05), while the expression levels of *PLIN5* and *SREBP1* decreased significantly (*p* < 0.05) (Figure 3D).

### 2.4. PLIN2 Interference Inhibits Hydrogen Peroxide-Induced Lipid Droplet Accumulation

In order to detect the effect of PLIN2 on hydrogen peroxide-induced lipid droplet formation, we designed a specific RNA interference fragment to interfere with PLIN2 expression in cells. RNA interference fragments of *PLIN2* were transfected into HepG2 cells using Lipofectamine 2000 (human negative interference fragments in the control group) and cells were further cultured for 48 h to ensure that PLIN2 expression was sufficiently affected. Cells were then treated with 200 nM hydrogen peroxide for 8 h. Intracellular lipid droplets were labeled using BODIPY 493/503 and were observed under a fluorescence microscope. The results showed that the lipid droplets in the interference group were significantly reduced compared with the control group (*p* < 0.05) (Figure 3E,F).

We detected the relative expression of PLIN2 to confirm the interference effect. The results showed that the expression level of PLIN2 was significantly decreased and the interference efficiency was higher than 70% (*p* < 0.05) (Figure 3G). We further examined the expression levels of *PLIN1*, *PLIN3*, *PLIN4*, *PLIN5*, *ATGL*, *SREBP1*, and *FSP27*. The results showed that the expression levels of *PLIN4*, *PLIN5*, *SREBF1*, and *FSP27* increased significantly (*p* < 0.05), while the expression levels of *PLIN1* and *PLIN3* decreased significantly (*p* < 0.05) (Figure 3H).

### 2.5. Hydrogen Peroxide Treatment Promotes the Formation of Lipid Droplets through Up-Regulating PLIN2

**Figure 4:**
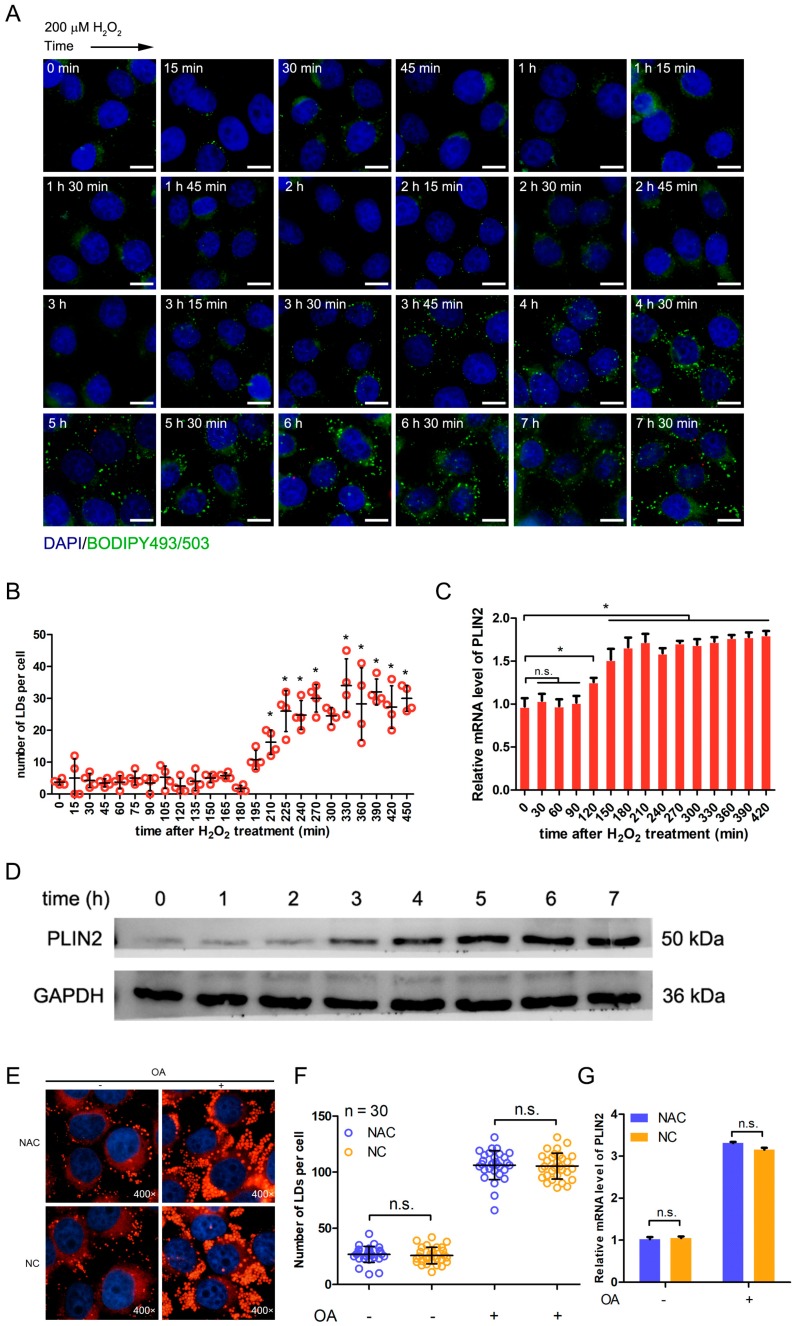
Hydrogen peroxide treatment promotes the formation of lipid droplets through up-regulating PLIN2. (**A**) The changes of cellular lipid droplets along with time after the treatment of 200 μM hydrogen peroxide. Scale bar, 20 μm. (**B**) Statistical analysis of the number of lipid droplets. *, *p* < 0.05. (**C**) The changes of PLIN2 expression along with time after the treatment of 200 μM hydrogen peroxide. *, *p* < 0.05. (**D**) Detection of PLIN2 protein expression level after the hydrogen peroxide treatment. (**E**) Detection of lipid droplets in cells treated with 1 mM NAC (N-Acetylcysteine) and 1 mM OA. (**F**) Statistical analysis of the number of lipid droplets. n.s., not significant. (**G**) Detection of expression of PLIN2 in cells treated with 1 mM NAC and 1 mM OA. n.s., not significant.

The expression of PLIN2 was important to the formation of lipid droplets that were induced by hydrogen peroxide treatment. It was not clear whether the increase of lipid droplets was caused by up-regulating of PLIN2. Therefore, we investigated the number of lipid droplets and the expression level of PLIN2 during the treatment of hydrogen peroxide from 0 to 420 min (Figure 4A–C). There was no significant difference in the number of lipid droplets before 210 min and then the number of lipid droplets significantly increased (*p* < 0.05) (Figure 4B). The expression level of PLIN2 was significantly up-regulated at 120 min after hydrogen peroxide treatment (*p* < 0.05) (Figure 4C). The results showed that the up-regulating of PLIN2 occurred earlier than the formation of lipid droplets after hydrogen peroxide treatment. (Figure 4B,C). Then, we analyzed the protein expression level of PLIN2 after the hydrogen peroxide treatment. The expression of PLIN2 protein was mildly increased at 2 h and was greatly increased at 3 h (Figure 4D). To further investigate the effect of ROS on the formation of lipid droplets and the expression of PLIN2, we detected these phenotypes using N-Acetylcysteine (NAC), which can reduce the level of cellular ROS. The presence of NAC did not change the number of cellular lipid droplets under normal or fatty acid (FA)-rich medium (Figure 4E,F). Moreover, the expression of PLIN2 was not changed by NAC (Figure 4G). The results showed that decreasing ROS level did not affect the formation of lipid droplets and the expression of PLIN2, whereas increasing ROS level up-regulated PLIN2 expression so that it promoted the formation of lipid droplets.

### 2.6. Exogenous Addition of Hydrogen Peroxide Promotes the Formation of Lipid Droplets in Murine Hepatocytes

**Figure 5:**
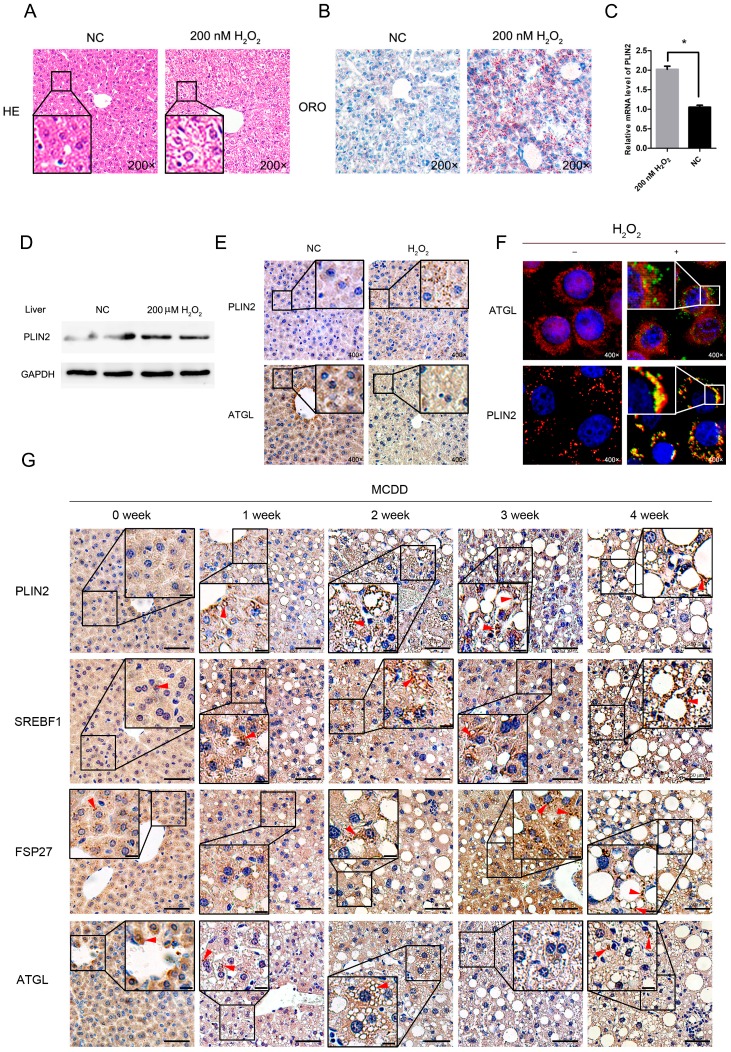
Following treatment with hydrogen peroxide, the expression level of PLIN2 in the liver tissue and the lipid content increased. Liver tissue morphology and lipid content were measured after intraperitoneal injection of 200 μM hydrogen peroxide for 24 h. (**A**) HE slices detection. (**B**) Oil Red O staining test. (**C**) The qRT-PCR detection of the mRNA expression level of PLIN2. *, *p* < 0.05. (**D**) Western blot detection of PLIN2 protein expression levels. (**E**) Immunohistochemical analysis of PLIN2 and ATGL in liver tissues treated with hydrogen peroxide. The magnification is 400×. (**F**) Cellular immunofluorescence analysis of PLIN2 and ATGL in cells treated with hydrogen peroxide. (**G**) Immunohistochemical analysis of PLIN2, SREBF1, FSP27, and ATGL in liver tissues of MCDD (methionine choline deficient diet) fed mice. The enhanced signals are indicated by red arrows. Sacle bar = 50 μm. Subfigure sacle bar = 10 μm.

To further investigate whether increased reactive oxygen species would lead to an increase in lipid droplets in the liver in vivo, we selected 6-week-old mice to be injected intraperitoneally with 500 μL of 200 μM hydrogen peroxide (formulated in 5% glucose solution). The control group was injected with an equal volume of 5% glucose solution. After 24 h, the liver tissue of the mice was taken for histological examination. The results of hematoxylin-eosin (HE) staining and Oil Red O staining showed that the number of lipid droplets in the liver tissue that was injected with hydrogen peroxide was significantly higher than that in the control group (Figure 5A,B). Then, we extracted total RNA and total protein from the mice liver tissues and detected the expression levels of related genes and proteins using real-time quantitative PCR and Western blot. The results showed that PLIN2 expression was up-regulated in the hydrogen peroxide treated group (Figure 5C,D). We then detected the localization of PLIN2 and ATGL in cells that were treated with hydrogen peroxide. A small number of lipid droplets existed in cells in the negative control (NC) group and localization of PLIN2 was mainly in cytoplasm. When treated with hydrogen peroxide, cells formed many lipid droplets and PLIN2 localized to the surface of lipid droplets (Figure 5E). The hydrogen peroxide treatment did not change the localization of ATGL in the cells (Figure 5F).

### 2.7. Lipid Droplet-Related Proteins Enhanced Expression in Steatotic Liver Tissue

To further assess whether these lipid droplet-related proteins enhanced expression in a steatotic process, we fed mice with methionine-choline-deficient diet (MCDD) to make a NAFLD phenotype in live tissue. We then detected the localization and expression levels of *PLIN2*, *ATGL*, *SREBF1*, and *FSP27* by the immunohistochemistry method. The results showed that the expression of all of these proteins was enhanced in the process of liver steatosis (Figure 5G). The localization of PLIN2 was on the surface of lipid droplets. FSP27 was mainly localized to the surface of lipid droplets and lipid droplet–lipid droplet contact sites. The localization of SREBF1 was mainly in cytoplasm, with a small portion in nucleus. The steatosis did not affect the localization of SREBF1. ATGL mainly localized to cytoplasm, with some targeted to the surface of lipid droplets. The excess lipid accumulation did not affect the localization of ATGL.

### 2.8. PLIN2 Modulates Lipid Droplet Formation in Cells via PPAR (Peroxisome Proliferator-Activated Receptor) and CREBBP (CREB Binding Protein) Signaling Pathways

**Figure 6:**
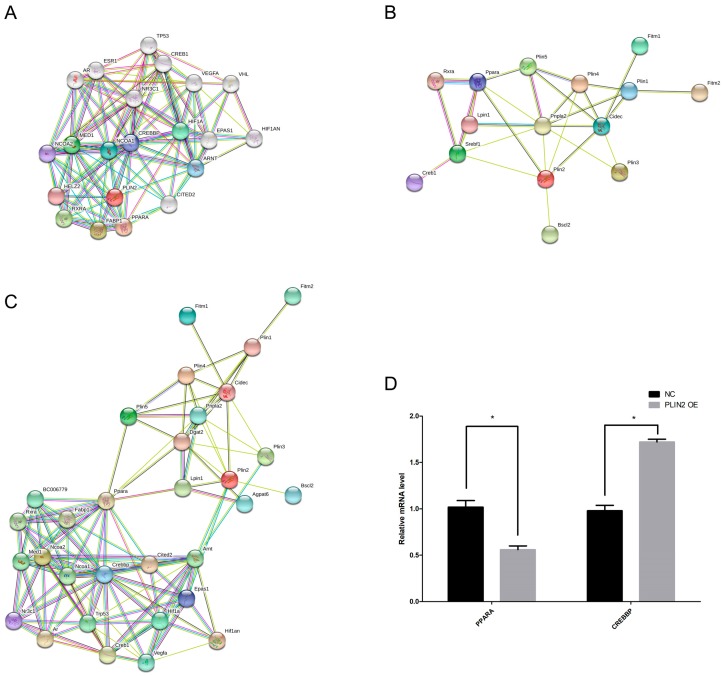
Protein interaction network analysis. (**A**) Analysis of proteins that interact with PLIN2. (**B**) Analysis of the interaction among PLIN2 and genes related to lipid droplet formation. (**C**) Comprehensive analysis of protein interaction networks. (**D**) Detection of mRNA expression levels of *PPAR*α and *CREBBP* (CREB binding protein) by qRT-PCR with the overexpression of *PLIN2*. *, *p* < 0.05.

Since PLIN2 overexpression promotes lipid droplet formation, we attempted to find proteins that co-express and interact with PLIN2 using the KEGG and STRING databases. The results indicated that the PLIN2 interaction protein network includes the following proteins: CITED2, NCOA2, NCOA1, HIF1A, TP53, ARNT, CREB1, VEGFA, RXRA, EPAS1, AR, CREBBP, MED1, FABP1, HELZ2, PPARα, HIF1AN, and NR3C (Figure 6A). According to the GeneCards database, these proteins are related to intracellular lipid metabolism. Among them, CREB1, RXRA, CREBBP, FABP1, and PPARα have important regulatory effects on intracellular lipid production and decomposition. We further selected proteins PLIN3, PLIN4, PLIN5, ATGL, SREBF1, FSP27, SEIPIN, FITM1, FITM2, LIPIN1, LIPIN2, and PLIN2, which are directly involved in lipid droplet formation and growth, to analyze the interactive relationship with PLIN2. The results showed that PLIN2 interacts with these proteins in one network (Figure 6B). We performed a comprehensive network analysis of the above proteins, and the results showed that these proteins exist in one interaction network (Figure 6C). In this interaction network, the most interacting proteins were with PPARα and CREBBP, indicating that these two proteins are at the core of this network (Figure 6C). Therefore, PLIN2 may affect the expression of lipid droplet-related proteins by affecting the expression of PPARα and CREBBP. We examined the effect of the overexpression of PLIN2 on the expression levels of PPARα and CREBBP by qRT-PCR. The results showed that overexpression of PLIN2 increased mRNA expression of CREBBP and decreased the mRNA expression of PPARα (Figure 6D).

## 3. Discussion and Conclusions

With the development of nonalcoholic fatty liver disease, the lipid content in hepatocytes increases and excessive fatty acid oxidative metabolism produces many by-products that contain a large amount of reactive oxygen species that are released into the whole cell via mitochondria. Previous studies have reported that there are high intracellular ROS in lipids [34]. As a potent oxidant, ROS plays a role of signaling molecules, binding and oxidizing various proteins, and regulating multiple signaling pathways, such as HSF1, NF-κB, p53, PI3K-Akt, and ERK-JNK-p38 [33]. It is well known that excessively high levels of ROS promote inflammation that is usually accompanied by the development of NAFLD. It can lead to the development of NASH and even liver fibrosis and cirrhosis, in some cases. However, whether lipid accumulation is disrupted due to elevated ROS levels disturbing intracellular lipid metabolism is currently unclear. Our results show that increasing intracellular ROS levels leads to the accumulation of intracellular lipid droplets, suggesting that elevated ROS levels disrupt the homeostasis of intracellular lipid metabolism. In order to investigate whether ROS promoted lipid droplet formation or inhibited the breakdown of lipid droplets and increased the number of lipid droplets, we examined several lipid droplet-related genes and found that *PLIN2*, *PLIN5*, *ATGL*, and *FSP27* expression levels were elevated with higher ROS levels (*p* < 0.05, Figure 2A). PLIN5 is a key factor in the contact of lipid droplets with mitochondria, which promotes the interaction between lipid droplets and mitochondrion, and increases the oxidative breakdown of neutral lipids in lipid droplets [21,22,23]. ATGL, a triglyceride enzyme, is an important regulator of lipolysis of lipid droplets that are localized on the surface of lipid droplets and those that are in contact with internal triglycerides and hydrolyzed [35,36]. An increase in the expression levels of these two genes means that the ability to decompose lipids in the cells is enhanced and, thus, ROS is less likely to promote lipid aggregation by inhibiting cell lipid breakdown. Increased expression levels of PLIN2 and FSP27 indicate enhanced intracellular lipid droplet formation, growth, and fusion. Therefore, ROS is likely to enhance the ability of intracellular lipid production, and the rate of formation is greater than the rate of decomposition, resulting in the aggregation of lipids.

Because PLIN2 is the first lipid droplet surface protein to be identified, it has been considered as the marker protein of lipid droplets. Its protein structure and subcellular localization are highly conserved in different species, which is essential for lipid droplet formation and morphological stability [37]. We selected PLIN2 for follow-up studies from lipid-related genes that were affected by ROS levels. We overexpressed PLIN2 in cells and examined the effect of PLIN2 on intracellular lipid droplets. The results showed that PLIN2 overexpression promoted intracellular lipid droplet formation (Figure 3A). We examined the localization relationship between PLIN2 and lipid droplets that were induced by ROS and found that PLIN2 is mainly surrounded by lipid droplets (Figure 2D). This result indicates that PLIN2 is essential for ROS-induced lipid droplet formation. To further demonstrate this indication, we interfered with the expression of PLIN2 in the cells and observed whether it affected the formation of lipid droplets. The results showed that the inhibition of PLIN2 expression reduced the number of lipid droplets in the cells, and the promotion of ROS to lipid droplets was weakened (Figure 3E). Therefore, PLIN2 plays an important role in the lipid droplet formation process. To further validate that hydrogen peroxide treatment induced lipid droplet formation via up-regulating PLIN2, we analyzed the time course of hydrogen peroxide-induced lipid droplet accumulation and PLIN2 expression. PLIN2 up-regulation occurred prior to lipid droplet accumulation (Figure 4). PLIN2 is considered as the marker of cellular lipid droplets, and indeed its expression is affected by the lipid content. It is well known that hydrogen peroxide treatment would induce more neutral lipid formation. In the present study, we considered that PLIN2 up-regulation by hydrogen peroxide treatment was the most important reason for the cellular lipid droplet accumulation. Experimentally, it is better to investigate the regulatory relationship between hydrogen peroxide treatment and PLIN2 expression under the condition of blocking lipid droplet formation. However, there is no useful specific pharmacological inhibitors blocking lipid droplet accumulation. Therefore, we tried to validate our hypothesis by the comparison of microscopy and qRT-PCR/Western blot data. Furthermore, we have optimized our experiment operation and settings to reduce the shortcomings of these traditional detection methods because detection of lipid droplets might be limited by the efficacy of the dye and microscope settings and qRT-PCR depends on various other parameters (cDNA, primer, etc.). The conclusion would be much more credible to perform this experiment using specific blocker of lipid droplet formation. We further validated the relationship among ROS, lipid droplet formation, and PLIN2 expression in vivo. After injection of 200 μM hydrogen peroxide (prepared in 5% dextrose solution), the lipid content in the liver tissue increased and the expression level of PLIN2 also increased (Figure 5). This result is the same as that in vitro. It indicates that elevated levels of ROS increase PLIN2 expression levels and promote lipid droplet formation, and aggregation is conserved between species.

Through expression and regulation network analysis, we can predict the expression and relationship between genes and can help to obtain signal regulation pathways. We analyzed the proteins interacting with PLIN2 and found that the proteins interacting with PLIN2 are related to intracellular lipid metabolism and that there are several important regulatory factors for lipid production and decomposition processes, such as PPARα, RXRA, CREB1, and CREBBP (Figure 6A). We further selected proteins that are involved in the lipid droplet formation process and performed protein interaction analysis with PLIN2. The lipid-drug-producing protein and PLIN2 interaction protein were analyzed comprehensively. It was found that PLIN2 interacted with FSP27, SREBF1, PLIN4, and SEIPIN and that these proteins could indirectly affect other lipid droplet-related proteins such as PLIN1, PLIN3, PLIN5, FITM1, FITM2, LIPIN1, LIPIN2, etc. (Figure 6B). We performed a comprehensive analysis of the proteins in the two networks and found that these proteins are mainly linked by the two protein complexes, PPARα-RXRA and CREB1-CREBBP (Figure 6C). Peroxisome proliferator-activated receptor (PPAR) is a major regulator of lipid metabolism, and PPARα is a free fatty acid receptor that plays a pivotal role in maintaining the homeostasis of lipid metabolism in the body. As the most active organ metabolism in the body, the liver is the junction and control center of endogenous and exogenous lipid metabolism pathways and is also the central target organ for anti-lipid peroxidation. Studies have shown that changes in PPARα activity are one reason for fatty liver occurrence and development [38,39,40,41]. CREB is a cAMP-reactive element binding protein—an important regulator in the nucleus of eukaryotic cells—and plays an important regulatory role in gene transcription, cell development and survival, and circadian rhythm. The cAMP/PKA signaling pathway plays a key role in the early stages of adipocyte differentiation and also plays an important physiological role in regulating fat cell differentiation by activating CREB phosphorylation [42]. In addition, CREB induces C/EBPβ expression during adipogenic differentiation, triggering cascade transcriptional responses, and thereby promoting the activation of C/EBPα, PPARγ and other important transcription factors [43]. Therefore, CREB is very important for the formation of intracellular lipids. CREB binding protein (CREBBP), as a transcriptional coactivator, is important to the function of CREB [44]. We found that the overexpression of PLIN2 decreased PPARα expression and increased CREBBP expression. PLIN2 can stabilize the lipid droplet morphology; hence, protecting the neutral lipid from hydrolysis by the lipase on the surface of the lipid droplet may affect the intracellular fatty acid content and, in turn, the expression and activation of PPARα. Increased expression of CREBBP promotes CREB transcriptional activity which, in turn, enhances CREB function. All the results were based on the bioinformatic analysis and more studies remain to be needed for protein-protein interaction in this progression through new methods such as asymmetric labeling and carbonyl-carbon selective heteronuclear NMR spectroscopy [45].

Many studies have reported that hydrogen peroxide treatment would up-regulate cellular lipid droplets content [7,8,10], especially in NAFLD [13,14]. For the progression of lipid droplet formation, PLIN2 is reported as a key regulator, which is usually regarded as a marker protein of lipid droplets [25,26,27,32]. Because the molecular mechanism of hydrogen peroxide-induced lipid droplet accumulation has not been totally clear, we assume that hydrogen peroxide-induced PLIN2 up-regulation induces lipid droplet accumulation. Besides supporting previous research, our study found that hydrogen peroxide-induced PLIN2 up-regulation occurred prior to lipid droplet accumulation, which indicated the importance of up-regulated PLIN2 to hydrogen peroxide-induced lipid droplet accumulation. Moreover, PLIN2 knockdown blocked hydrogen peroxide-induced lipid droplet formation. Our study mainly identified a correlation between H2O2-induced PLIN2 and lipid droplet up-regulation, and more studies are needed to reveal the regulatory role of ROS in the development of NAFLD.

In conclusion, elevated levels of reactive oxygen species promoted the expression of PLIN2, while elevated PLIN2 expression affects the PPARα-RXRA and CREB-CREBBP signaling pathways, thereby affecting the homeostasis of intracellular lipid metabolism and promoting the accumulation of intracellular lipids.

## 4. Methods and Materials

### 4.1. Animals and Cell Lines

Six-week-old c57/bl6 male mice were purchased from Hubei Center for Disease Control and Prevention. All mice were housed in a normal environment and were provided with food and water. The methods were carried out in accordance with the approved guidelines from Huazhong Agricultural University and the scientific, ethical, and legal principles of the Hubei Regulations for the Administration of Affairs Concerning Experimental Animals. All of the experimental protocols were subject to approval by the Ethics Committee of Huazhong Agricultural University (HZAUMU2013-0005). The HepG2 cell line was gifted by the lab of Prof. Xianghua Yan, Huazhong Agricultural University (Wuhan, China).

### 4.2. Antibodies

Rabbit polygonal antibodies that were used included anti-GAPDH (#CSB-PA00025A0Rb Flarebio Biotech LIC., Wuhan, China), anti-ADRP/perilipin2 (#15294-1-AP, Proteintech, Chicago, IL, USA). The following secondary antibodies were utilized: Alexa Fluor 555-labeled Donkey Anti-Rabbit IgG (H+L) (#A0453, Beyotime, Shanghai, China) and HRP-labeled Goat Anti-Rabbit IgG (H+L) (#GB23303-1, Servicebio, Wuhan, China).

### 4.3. Plasmid DNA Construction

Full-length coding sequence (CDS) sequences encoding PLIN2 (NM_001122.3) were amplified using a cDNA library of HepG2 cells and were subcloned into the pcDNA3.1 vector (gifted from the lab of Prof. Dequan Xu, Huazhong Agricultural University).

### 4.4. Cell Culture and Transfection

HepG2 cells were cultured in Dulbecco’s Modified Eagle Medium (DMEM) (HyClone) with 10% fetal bovine serum (AusGeneX, Molendinar, Australia), 100 unit/mL penicillin, and 100 μg/mL streptomycin in dishes at 37 °C and were transfected with Lipo6000™ Transfection Reagent (#C0528, Beyotime). HepG2 cells were seeded on the cell slide in a 6-well plate and were transfected with the plasmid vector according to the transfection reagent instructions. For oleic acid treatment, a 20 mM oleic acid-phosphate buffer saline (PBS) mixture and 20% FA-free bovine serum albumin (BSA) medium were prepared and both media were heated in a 70 °C water bath for 30 min. Finally, the media were mixed. The 10-mM oleic acid-BSA mixture was added to the cell cultural medium at 1:49 (*v*:*v*). The cells that were seeded on the slides or plates were washed 3 times using PBS, 1 mL oleic acid medium was added to the well, and the cells were cultured for 12 h.

### 4.5. Hydrogen Peroxide Treatment

Due to the molarity of 30% hydrogen peroxide (i.e., 10 M), it was diluted 10,000× by DMEM medium to 1 mM concentration after the medium was sterilized using a 0.22 μm filter. The hydrogen peroxide was then diluted to different concentrations and the medium was used to treat cells. Cells have many kinds of superoxide dismutase that could decrease hydrogen peroxide; therefore, we used 1 mM hydrogen peroxide to treat the cells for 30 min in order to eliminate the effect of superoxide dismutase. The cells were then washed three times using PBS and were treated with different concentrations of hydrogen peroxide media; this operation is important for the treatment of hydrogen peroxide, especially if in low concentrations. Due to the significant impacts of small amounts of metals, such as iron and copper, on the outcomes of in vitro experiments, the medium contained ferric nitrate·9 H_2_O (0.1 mg/L). No other iron or copper was present. The water that was used in this experiment was double distilled and deionized.

### 4.6. Hematoxylin-Eosin Staining and Oil Red O Staining of Histological Sections

For HE staining, the liver tissues were divided and fixed in 4% paraformaldehyde for 24 h. After dehydration, the tissues were embedded in paraffin for sectioning. After the dewaxing process, the sections were stained with hematoxylin for 8–10 min. Sections were dehydrated again and were stained with eosin for 3 min. After dehydration, the sections were sealed with neutral gum. For Oil Red O staining, the liver tissues were fixed in 10% formalin and were prepared for frozen sections at a thickness of 6–10 μm. The sections were mounted on slides and were dried for 10 min at room temperature. The sections were incubated in 75% alcohol for 10 s and were stained in Oil Red O solution (#G1016, Servicebio). The slides were differentiated for 1 min until the background was colorless. After washing for 2 min, the sections were stained with hematoxylin for 8 min. The sections were washed and differentiated in a 1% aqueous HCl solution for 1 min. The sections were incubated in ammonia for several minutes. The slides were sealed with a glycerin gelatin solution.

### 4.7. Lipid Droplets Marking and Observation

Cells were seeded on slides in a 24-well plate and were cultured for 24 h. The slides were fixed in 4% paraformaldehyde for 15 min at room temperature. The slides were stained with BODIPY 493/503 (#D3922, Invitrogen, Carlsbad, CA, USA) for 45 min at 37 °C and were then stained with DAPI (#G-1012, Servicebio) for 10 min at 37 °C. After washing three times with PBS for 10 min each, the slides were sealed with an anti-fluorescent quenching solution for microscopic observation.

### 4.8. Western Blot and Real-Time PCR

Real-time PCR was performed using the QuantStudio 6 Flex Real-Time PCR System (ABI, Thermo Fisher, Shanghai, China) and the following PCR program: denaturation at 95 °C for 2 min; amplification for 40 cycles at 95 °C for 10 s; annealing and extension at 60 °C for 32 s. Primer sequences are shown in Table 1. Specific amplification for certain PCR reactions was assessed using a melting curve. One negative control reaction, in which the cDNA template was replaced by water, was performed to avoid potential contamination. The sample from each well was repeated three times, and the comparative *C*t (ΔΔ*C*t) value method was used for relative quantification. GAPDH (NM_002046.6) was used as the reference gene.

Transfected cells were homogenized in 1 mL of 25 mM Tris/1 mM ethylenediaminetetraacetic acid pH buffer (pH 7.5). Homogenates were separated by 12.5% sodium dodecyl sulfate-polyacrylamide gel electrophoresis (SDS-PAGE) and were transferred to a polyvinylidene fluoride (PVDF) membrane (Millipore, Bedford, MA, USA) using a semidry electrophoretic method. The blocked membranes (5% BSA in tris-buffered saline (TBS) buffer containing 0.1% Tween 20) were incubated with antibodies overnight at 4 °C. The blots were extensively washed three times with tris-buffered saline with Tween 20 (TBST) buffer for 10 min and were incubated under gentle agitation with the secondary antibodies for immunodetection. The antigen–antibody reaction was incubated for 1 h, and the cross-reacting proteins were detected. Pre-stained molecular weight markers 10–170 kD in weight (Fermentas, Burlington, ON, Canada) were used as standards.

### 4.9. Immunofluorescence Assay

Cells on the slides were fixed in 4% paraformaldehyde for 15 min at room temperature. The paraformaldehyde was removed, and the cells were washed with PBS three times for 10 min each. The slides were permeabilized using 0.5% Triton X-100 for 40 min at 37 °C. Next, the 0.5% Triton X-100 was removed and the cells were again washed with PBS three times for 10 min each. Using a 3% BSA solution, the slides were blocked for two hours at 37 °C. The blocking solution was removed, and the primary antibody was diluted in the first solution (#NKB-301, Can Get Signal Immunoreaction Enhancer Solution, TOYOBO, Shanghai, China) was added to the cell wells for a 16 h incubation at 4 °C. The primary antibody solution was removed, and the slides were washed with PBS three times for 10 min each. The second antibody diluted in a second solution (#NKB-301, Can Get Signal Immunoreaction Enhancer Solution, TOYOBO) was added to the cell wells and was incubated for 1 h at 37 °C. The slides were washed with PBS three times for 10 min each. The slides were stained with BODIPY 493/503 (#D3922, Invitrogen) for 45 min at 37 °C and were then stained with DAPI (#G-1012, Servicebio) for 10 min at 37 °C. After washing with PBS three times for 10 min each, the slides were sealed with an anti-fluorescent quenching solution. An OLYMPUS BX53 was used for microscopic observation of the slides and a DP80 camera was used to capture cell images. The images were analyzed using Cell Sense software (Cell Sense Standard version, OLYMPUS, Tokyo, Japan) and Photoshop CS6 (Adobe, San Jose, CA, USA).

### 4.10. Intraperitoneal Injection of Hydrogen Peroxide in Mice

6-week-old c57/bl6 male mice were divided into two groups of three mice each. One group was injected intraperitoneally with 500 μL of 200 nM hydrogen peroxide (prepared in 5% dextrose solution) and the other group was injected intraperitoneally with an equal volume of 5% dextrose solution. After 24 h, the liver tissues from the two groups of mice were taken for histomorphological examination.

### 4.11. Immunohistochemistry Assay

The hepatic tissues with steatosis were collected from mice that were fed a methionine choline deficient diet (MCDD). Paraffin sections were placed in an oven at 67 °C, were baked for 2 h, dewaxed to water, and were rinsed three times with PBS (pH 7.4) for three minutes each time. The dewaxed hydrated tissue section was then placed on a high temperature plastic slice rack, which was in turn placed in citrate buffer (pH 6.0) that had been brought to boiling using a microwave. The tissue section was treated in the microwave for 10 min before being removed and allowed to drain naturally. The slide was removed from the buffer, rinsed twice with distilled water, and was then rinsed three times with PBS. One section of 3% H_2_O_2_ was added to each section and was incubated for 10 min at room temperature to block endogenous peroxidase activity; it was then rinsed with PBS three times. After removing the PBS solution, one drop of corresponding primary antibody (corresponding to the dilution factor) was added to each section, which was then incubated for 2 h at room temperature. The sections were again rinsed in PBS three times. The PBS solution was removed, one drop of polymer enhancer was added to each section, and the sections were incubated for 20 min at room temperature, before again being rinsed three times in PBS. The PBS solution was removed and 1 drop of the enzyme-labeled anti-mouse/rabbit polymer was added to each section, which was then incubated for 30 min at room temperature. Sections were again rinsed three times in PBS. PBS solution was removed and one drop of freshly prepared DAB (diaminobenzidine) solution was added to each section prior to microscope observation for 5 min. The degree of dyeing is under the microscope. Then, the section was washed by PBS for 10 min. Sumu was dyed for 2 min and then using hydrochloric acid differentiation. Then, the section was washed by tap water for 10–15 min. The following were performed: dehydration, transparency, sealing, and examination.

### 4.12. Bioinformatics and Data Analysis

The protein–protein interaction network was analyzed by STRING (https://string-db.org/), gene information was obtained from GeneCards (https://www.genecards.org/), and the signaling pathway analysis was undertaken using the KEGG pathway (https://www.kegg.jp/).

### 4.13. Statistical Analyses

All of the experiments were repeated three times. Data were extracted as the mean ± SD. The Student’s *t*-test was used for statistical comparisons and a *p*-value < 0.05 was considered statistically significant.

## Authors Contribution

Y.J. designed this work and wrote the article. Y.T. performed part of the experiments. L.C. did the statistical analysis of the lipid droplets in the figures. Y.L. helped with the interactive network analysis. All of the authors read and approved the final manuscript. Z.R. designed this work and participated in the revision of this article. 

## Figures and Tables

**Table 1:** Primer used for SYBR Green I qRT-PCR validation.

Gene Symbol	Primer Sequence 5′-3′	Product Size (bp)	Accession
*PLIN1 (perilipin1)*	Forward: TGGGTGGTGTGGCACATAC	143	XM_005254934.4
	Reverse: CCTCCCCTTGGTTGAGGAGA		
*PLIN2 (perilipin2)*	Forward: TTGCAGTTGCCAATACCTATGC	148	XM_017014259.2
	Reverse: CCAGTCACAGTAGTCGTCACA		
*PLIN3 (perilipin3)*	Forward: GCCCAAGAGATGGTGTCTAGC	119	NM_005817.4
	Reverse: CCGGTCACTACGGACTTTGT		
*PLIN4 (perilipin4)*	Forward: GGAGCTGCAACCTTCGGAAA	131	NM_001080400.1
	Reverse: GGACCACTCCCTTAGCCAC		
*PLIN5 (perilipin5)*	Forward: AAGGCCCTGAAGTGGGTTC	192	NM_001013706.2
	Reverse: GCATGTGGTCTATCAGCTCCA		
*SREBP1 (sterol regulatory element binding protein 1c)*	Forward: ACAGTGACTTCCCTGGCCTAT	222	XM_024450893.1
	Reverse: CATGGACGGGTACATCTTCAA		
*FSP27*	Forward: ATTGATGTGGCCCGTGTAACG	122	NM_001199623.1
	Reverse: CAGCAGTGCAGATCATAGGAAA		
*ATGL*	Forward: ATGGTGGCATTTCAGACAACC	89	NM_020376.3
	Reverse: CGGACAGATGTCACTCTCGC		
*CREBBP*	Forward: CGGCTCTAGTATCAACCCAGG	237	NM_004380.2
	Reverse: TTTTGTGCTTGCGGATTCAGT		
*PPAR* *α*	Forward: TTCGCAATCCATCGGCGAG	146	NM_005036.5
	Reverse: CCACAGGATAAGTCACCGAGG		
*GAPDH*	Forward: CTGGGCTACACTGAGCACC	101	NM_002046.6
	Reverse: AAGTGGTCGTTGAGGGCAATG		

## Abbreviations

ROS	reactive oxygen species
NAFLD	non-alcoholic fatty liver disease
NASH	non-alcoholic steatohepatitis
PLIN1	perilipin1
PLIN2	perilipin2
PLIN3	perilipin3
PLIN4	perilipin4
PLIN5	perilipin5
FSP27/CIDEC	cell death-inducing DFFA-like effector c
SREBF1	sterol regulatory element binding transcription factor 1
SREBP1c	sterol regulatory element binding protein 1c
PPAR	peroxisome proliferator activated receptor alpha
RXRA	retinoid X receptor alpha
CREB	cAMP responsive element binding protein
CREBBP	CREB binding protein
SOD1	superoxide dismutase 1
JNK/MAPK8	mitogen-activated protein kinase 8
AMPK	adenosine 5‘-monophosphate (AMP)-activated protein kinase
OA	oleic acid
CITED2	Cbp/P300 interacting transactivator with Glu/Asp rich carboxy-terminal domain 2
NCOA1	nuclear receptor coactivator 1
NCOA2	nuclear receptor coactivator 2
HIF1	hypoxia inducible factor 1
HIF1AN	hypoxia inducible factor 1 alpha subunit inhibitor
TP53	tumor protein p53
ARNT	aryl hydrocarbon receptor nuclear translocator
VEGFA	vascular endothelial growth factor A
EPAS1	endothelial PAS domain protein 1
AR	androgen receptor
MED1	mediator complex subunit 1
FABP1	fatty acid binding protein 1
HELZ2	helicase with zinc finger 2
NR3C	nuclear receptor subfamily 3 group C member
SEIPIN/BSCL2	seipin lipid droplet biogenesis associated
FITM1	fat storage inducing transmembrane protein 1
FITM2	fat storage inducing transmembrane protein 2
ATGL/PNPLA2	patatin like phospholipase domain containing 2
